# Anxiolytic Effects of Repeated Cannabidiol Treatment in Teenagers With Social Anxiety Disorders

**DOI:** 10.3389/fpsyg.2019.02466

**Published:** 2019-11-08

**Authors:** Nobuo Masataka

**Affiliations:** Primate Research Institute, Kyoto University, Inuyama, Japan

**Keywords:** cannabidiol, social anxiety disorder, cannabis, cannabinoid, social phobia, avoidant personality disorder, social withdrawal

## Abstract

Accumulated evidence indicates that cannabidiol (CBD), a nonpsychotomimetic and nonaddictive main component of the *Cannabis sativa* plant, reverses anxiety-like behavior. The purpose of the present study was to assess the efficacy of CBD treatment for Japanese late teenagers with social anxiety disorder (SAD). Thirty-seven 18–19-year-old Japanese teenagers with SAD and avoidant personality disorder received, in a double-blind study, cannabis oil (*n* = 17) containing 300 mg CBD or placebo (*n* = 20) daily for 4 weeks. SAD symptoms were measured at the beginning and end of the treatment period using the Fear of Negative Evaluation Questionnaire and the Liebowitz Social Anxiety Scale. CBD significantly decreased anxiety measured by both scales. The results indicate that CBD could be a useful option to treat social anxiety.

## Introduction

The primary noneuphorizing and nonaddictive compound of cannabis, cannabidiol (CBD), has recently been shown to possess considerable therapeutic potential for treating a wide range of neuropsychiatric disorders (De Gregorio et al., 2019). They include chronic pain (Costa et al., 2007), nausea (Parker et al., 2006), epilepsy (Devinsky et al., 2016), psychosis (McGuire et al., 2018), and anxiety (Scuderi et al., 2009; Whiting et al., 2015). CBD in therapeutics is used within a large therapeutic window, which ranges from 2.85 to 50 mg/kg/day (Whiting et al., 2015; Devinsky et al., 2016). While this fact indicates that its therapeutic dose is still mostly unknown, clinical studies have revealed that CBD could produce analgesic and anxiolytic effects exerted through its interaction with 5HT1A receptors (De Gregorio et al., 2019). As a potential anxiolytic treatment, in particular, it has drawn increasing interest. A review (Blessing et al., 2015) concluded that existing preclinical evidence strongly supports CBD as a treatment for generalized anxiety disorder, panic disorder, obsessive-compulsive disorder, and posttraumatic disorder when administered acutely.

Clinical data showing therapeutic effects of CBD in patients with anxiety disorders, however, are still meager (Bergamaschi et al., 2011; Crippa et al., 2011). The purpose of the present study was to investigate these effects in patients with social anxiety disorder (SAD).

SAD is characterized by excessive anxiety in situations where a person might feel judged, such as performance situations, and situations involving interpersonal contact with others (American Psychiatric Association, 2000). This is the fear of social situations that may cause humiliation or embarrassment. While it is one of the common anxiety disorders (Craske et al., 2017), it is a relatively new area of research, and thus, its etiology, effects, and treatment are not clearly understood. Even prevalence rates reported in the literature vary across studies (Antony and Rowa, 2008). For instance, lifetime prevalence estimates for SAD based on large community samples in the United States range from 3 to 13%. In addition, some studies report that SAD has a higher incidence in females than in males (Kessler et al., 2005).

Developmentally, SAD is likely to not only begin in adolescence (mid to late teens) but can also occur earlier in childhood (Somers et al., 2006). A significant number of adults report that they have had problems with social anxiety for their entire lives or as long as they can remember (Brown et al., 2001; Masataka, 2003). A large-scale study of individuals presenting at an anxiety clinic found a mean age of onset of 15.7 years, a number that was younger than the onset of other anxiety disorders (Merikangas et al., 2011).

SAD is best treated with psychotropic medication and cognitive behavioral therapies (CBT), and the most effective treatments are a combination of both (Nordahl et al., 2016). They consist of monoamine oxidase inhibitors, the serotonin reuptake inhibitors, benzodiazepines, and individual cognitive behavioral therapy. CBT typically includes 10–15 weekly sessions and consists of a variety of strategies, such as self-monitoring, psychoeducation, cognitive therapy, exposure-based techniques, and social skills training. While this method has been proven to be effective for SAD if it is executed, it is also true that people with the disorder quite often show unwillingness (Ryan and Warner, 2012) to receive CBT. In fact, a study reported that 92% of individuals with SAD expressed concerns about starting treatment and that is the biggest barrier to treatment that should be overcome (Kessler et al., 2005).

In this regard, preliminary findings reported by a study (Bergamaschi et al., 2011) that investigated the efficacy of CBD with patients with SAD are noteworthy. In that study, 12 patients with SAD were provided with a single dose of CBD (600 mg). When the anxiety induced by simulated public speaking was compared between pretreatment and posttreatment, its level showed a significant decrease after the treatment, whereas no such change was observed in the placebo group of 12 other patients.

The purpose of the current study was to pursue this issue further and to investigate the possible efficacy of CBD as at least an adjunctive option for intervention in people with SAD. While SAD has been classified into several subtypes so far (Antony and Rowa, 2008), here, the author concentrated the research on that with avoidant personality disorder because this subtype is the most commonly diagnosed and is becoming a serious social problem in Japan, where the current study was conducted (Ogino, 2004; Saito, 2007; Teo, 2010). The author attempted to systematically assess the efficacy of CBD in a total of 37 18–19-year-old Japanese with SAD, all of whom had been naive to any form of treatment, by measuring the level of the symptoms of SAD with both the Fear of Negative Evaluation Questionnaire (FNE; Watson and Friend, 1969) and the Liebowitz Social Anxiety Scale (LSAS; Liebowitz, 1987) using an exploratory double-blind parallel-group trial experimental paradigm.

FNE is a 30-item measure of apprehension and anxiety over anticipated social evaluations. This measure uses a true-false scale and is known to show good internal consistency and test-retest reliability (Watson and Friend, 1969). FNE has a range from 0 to 30, with high scores indicating higher levels of social anxiety. LSAS is a short questionnaire to assess the range of social interaction and performance situations feared by a person in order to assist in the diagnosis of SAD (Liebowitz, 1987). It has been commonly used to investigate outcomes in clinical trials and, more recently, to evaluate the effectiveness of psychological treatments (De Gregorio et al., 2019). The scale features 24 items, which are divided into two subscales. Thirteen questions relate to performance, and 11 relate to situations.

The author attempted to assess the efficacy of CBD by comparing the FNE and the LSAS scores measured before the commencement of the 4-week-long intervention (preintervention) and the scores measured after the completion of the intervention (postintervention) in the group of participants who were provided with CBD (the CBD group) and in the group whose participants were provided with placebo (the placebo group). The author hypothesized that CBD would significantly decrease anxiety measured by both of the two scales employed.

## Materials and Methods

### Ethics Statement

This investigation was conducted according to the principles expressed in the Declaration of Helsinki. All experimental protocols were consistent with the Guide for Experimentation with Humans and were approved by the Institutional Ethics Committee of Kyoto University (#2018-150), the regional committee for medical and health research ethics (#2018/1783), and the Japanese Data Inspectorate for clinical trials (JCT0018004564). The author obtained written informed consent from all of the participants involved in the study. Written informed consent was additionally obtained from the parent/legal guardian of all participants who were younger than the age of consent at the time of the study.

### Design and Participants

A randomized, placebo-controlled, comparative study with a total of 37 Japanese adolescents was undertaken. Double masking was conducted, and the participants and the investigator were blinded regarding which condition (CBD oil or placebo) under which each participant was studied.

At the commencement of the study, 40 teenagers with SAD participated, and 20 of them were assigned to the CBD group and the other 20 to the placebo group. This sample size was determined because the current study had been approved by the ethics committee on the condition that, as a pilot study, no more than 20 teenagers take CBD oil. Of the 40 participants, three in the CBD group declined daily treatment with CBD oil during the study because they disliked the smell and the taste of the oil.

In all, 26 males and 11 females 18–19-year olds were included in the study (12 males and 5 females for the CBD group and 14 males and 6 females for the placebo group). They were all naive to cannabis and diagnosed by psychiatrists in several hospitals located in the vicinity of Osaka Prefecture, Japan, using the Structured Clinical Interview for DSM-IV Axis I Disorders (SCID-I/P; Di Nardo et al., 1994) and Axis II Personality Disorders (First et al., 1997). For all of the participants, symptoms had lasted for at least 6 months at the commencement of the study. Exclusion criteria were experience of receiving previous or concurrent psychological or drug treatment, any form of psychotic or organic illness, diagnosis of cluster A or B personality disorder, acute suicidality, and drug and alcohol dependence. They have not had a comorbid diagnosis of other anxiety or mood disorders.

### Assessment

The participants were invited to attend an assessment interview for possible participation in the present study. None of them were under CBT. Assessment of all of them was undertaken by psychiatrists who were trained in administering the Structured Clinical Interview for DSM-IV (SCID I and II; Liebowitz, 1987; Di Nardo et al., 1994; First et al., 1997). The participants completed the first battery of self-report measures before attending the assessment interview. The baseline period for them was a minimum of 3 weeks showing stable FNE scores (Watson and Friend, 1969; the primary outcome measure). Subsequently, after the assessment interview, they rated themselves on the FNE and LSAS (Liebowitz, 1987) over the succeeding weeks (preintervention). All of the participants had a stable FNE score over the 3 consecutive weeks and were therefore scheduled for intervention within a week after the third-baseline measuring point.

When the 4-week-intervention ended, the FNE and LSAS were completed again by each participant (postintervention), and their scores were compared with those recorded at preintervention. Then, the SCID I and II were administered again by the same psychiatrists who met the participants at preintervention.

After the completion of the intervention, at the follow-up, the clinical psychologists who had been responsible for the intervention visited the participants briefly at their home once a week to check for any effect of it on their health. This follow-up continued for up to a 6-month-period.

### Intervention

All of the participants were randomly assigned into either the CBD group, in which they were to daily receive 300 mg of CBD administered in a single dose in the afternoon, or the placebo group, in which they were to daily receive a matching placebo. The CBD dose was based on that used in the first study showing acute anxiolytic effects in healthy subjects exposed to a simulated public speaking test (Zuardi et al., 1993). This assignment was conducted by an independent statistician who did not know about the purpose of the present research. The CBD used was RSHO-X Hemp Oil (the product of HempMeds, USA) that was produced from the stalk of hemp plants. A 236-ml bottle of the product that was for sale by the company contained 5,000 mg of CBD (21.4 mg/ml) but no delta-9-tetrahydrocannabinol (THC). It did not contain other cannabinoids or terpenes.

The placebo contained olive oil. The CBD oil containing 300 mg of CBD or the equivalent amount of the placebo was administered orally to each of the participants of the CBD group and each of the participants of the placebo group, respectively. For each participant, roughly 420 ml of the CBD oil or the same amount of the placebo was rebottled in a container that was different from that in which it had been originally bottled and that was identical in size and color as well as appearance to the oil administered to the other group.

The container was provided to a clinical psychologist who was employed by the principal investigator and was predetermined to be responsible for each participant. The psychologist who did not know whether the bottle contained CBD or not visited the home of the participant with the container every afternoon and administered the necessary amount of the prepared oil to the participant, using a syringe, during the 4-week intervention period.

While CBD oil had a characteristic smell and taste, all of the psychologists and the participants had been naive to the CBD oil as well as the placebo. The interview with them that was conducted after the completion of the study revealed that none of them noticed the difference between the two.

## Results

**Figure 1:**
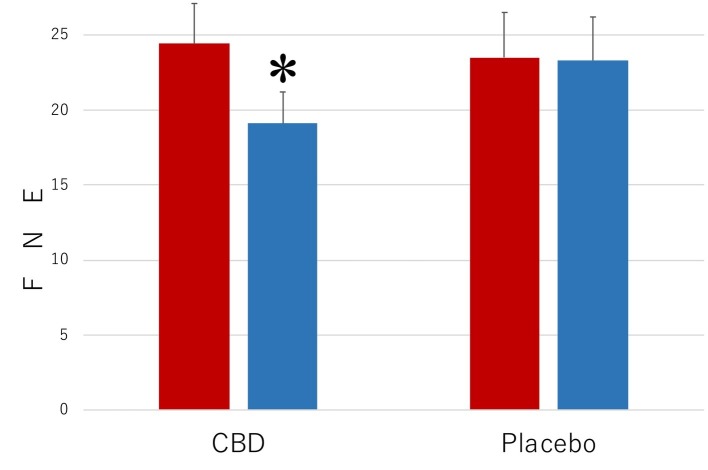
Scores of Fear of Negative Evaluation Questionnaire (FNE) in the participants who received the intervention with cannabidiol (CBD; *n* = 17) and in the participants who received the intervention with placebo (Placebo; *n* = 20). The participants were evaluated before (Pre) and after (Post) treatment. Error bars represent SDs. * indicates significant difference from pretreatment measurement.

The results of the measurements with FNE of the level of the symptoms that were associated with SAD are shown in Figure 1. When the collected data were analyzed by a 2 (period of measurements: preintervention versus postintervention, MEASUREMENT) × 2 (participant group: the CBD group versus the Placebo group, PARTICIPANT) repeated-measures ANOVA (analysis of variance), the main effect was statistically significant for MEASUREMENT (*F*_1,35_ = 10.35, *p* = 0.003, $\eta_{p}^{2}$ = 0.0228) but not for PARTICIPANT (*F*_1,35_ = 2.69, *p* = 0.11, $\eta_{p}^{2}$ = 0.071). The interaction between these factors was significant (*F*_1,35_ = 44.81, *p* < 0.001, $\eta_{p}^{2}$ = 0.561). The mean FNE score (SD) of the CBD group was 24.4 (2.7) in the preintervention measurement and 19.1 (2.1) in the postintervention measurement and that of the placebo group was 23.5 (2.1) in the preintervention measurement and 23.3 (2.9) in the postintervention measurement.

Subsequent analyses of simple main effects (using Bonferroni correction), which were performed because of the significant interactions between MEASUREMENT and PARTICIPANT, revealed that the mean score of the CBD group was lower in the postintervention measurement than in the preintervention measurement (*p* = 0.02), while no such difference was found in the placebo group (*p* = 0.29). Scores of the participants in the CBD group were lower than those of the placebo group in the postintervention measurement (*p* = 0.0002), but the scores were not statistically significantly different from one another in the preintervention measurement (*p* = 0.71).

**Figure 2:**
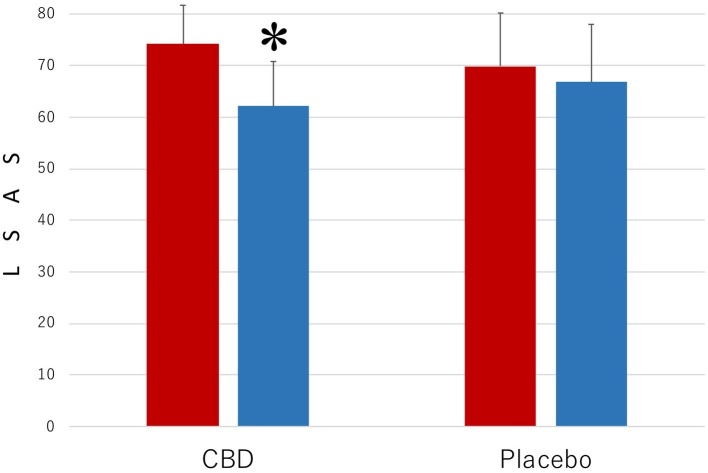
Scores of Liebowitz Social Anxiety Scale (LSAS) in the participants who received the intervention with cannabidiol (CBD; *n* = 17) and in the participants who received the intervention with placebo (Placebo; *n* = 20). The participants were evaluated before (Pre) and after (Post) treatment. Error bars represent SDs. * indicates significant difference from pretreatment measurement.

Figure 2 presents the results of the measurements with LSAS of the level of the symptoms that are associated with SAD. The results were strikingly similar to those shown in Figure 1. The main effect was statistically significant for MEASUREMENT (*F*_1,35_ = 10.35, *p* = 0.003, $\eta_{p}^{2}$ = 0.023) but not for PARTICIPANT (*F*_1,35_ = 0.45, *p* = 0.57, $\eta_{p}^{2}$ = 0.011). The interaction between these factors was significant (*F*_1,35_ = 39.16, *p* < 0.001, $\eta_{p}^{2}$ = 0.528). The mean LSAS score (SD) of the CBD group was 74.2 (7.5) in the preintervention measurement and 62.1 (8.7) in the postintervention measurement and that of the placebo group was 69.9 (10.3) in the preintervention measurement and 66.8 (11.2) in the postintervention measurement.

Another *post hoc* test revealed that the mean LSAS score of the CBD group was lower in the postintervention measurement than in the preintervention measurement (*p* = 0.03), but no such difference was found in the placebo group (*p* = 0.42). Scores of the participants in the CBD group were smaller than those of the placebo group in the postintervention measurement (*p* = 0.0018), but the scores in the two groups were not statistically significantly different from one another in the preintervention measurement (*p* = 0.66).

At the follow-up conducted after the completion of the intervention, none of the participants had any significant health complaint, although no systematic evaluation of side effects was conducted. At that time, among the 17 participants included in the CBD group, nine reported that they decided to receive some form of treatment (medication and CBT) by regularly visiting hospitals, while none of the 20 participants in the control group was found to make such a decision. The participants of the CBD group were more likely to make such decision than those of the placebo group were (*χ*^2^(1) = 13.99, *p* < 0.001).

## Discussion

The anxiolytic effects of CBD have been extensively demonstrated in animal studies and in healthy volunteers subjected to anxiety induced by several procedures, including the simulation of public speaking (Zuardi et al., 1993; Blessing et al., 2015). A pioneering study that investigated the effects on SAD patients showed that CBD reduces anticipatory anxiety (Crippa et al., 2011). Moreover, CBD was found to exert a significant effect on increased brain activity in the right posterior cingulated cortex that is thought to be involved in the processing of emotional information. A subsequent study (Bergamaschi et al., 2011) experimentally demonstrated a reduction in the anxiety provoked by simulated public speaking by a single dose of CBD in patients with SAD, although the findings were preliminary. Based on these findings, the current study was conceived to extend the published research into a more systematic study on the effect of CBD on teenagers with SAD with avoidant personality disorder for a longer period. Its results are consistent with those obtained by the previous research and indicate that intervention with CBD for a 4-week period reduced the level of symptoms in teenagers with SAD, as measured by FSE and LSAS.

As an option for medication treatment for SAD, so far, the use of paroxetine has been reported to be most effective (Nordahl et al., 2016). That study reported that a 26-week daily treatment with paroxetine alone produced a 5.2-point decrease in the FNE score and a 10.2-point decrease in the LSAS score. Those reported decreases in symptoms were almost equivalent to the observed decreases induced by CBD here, although the treatment groups studied in the two studies were not closely compatible.

In children and adolescents, SAD is known to be among the most common mental disorders (De Gregorio et al., 2019). A survey conducted in the United States showed that the disorder starts as early as age 5 and peaks around age 12 (Merikangas et al., 2011). When untreated, it runs a chronic course into adolescence and eventually adulthood. In Japan, notably, the population of such teenagers with avoidant personality disorder who “seclude themselves for more than six months at home” (Saito, 2007) and “typically withdraw from most social activities and retreat into their living spaces” (Teo, 2010) is estimated to have reached 1,000,000 (Ogino, 2004), and this has become a serious social problem. When they are provided with a higher level of social support, their quality of life (QOL) is likely to increase, whereas it deteriorates with poor support. The teenagers with SAD in Japan who have higher levels of social withdrawal along with such poor support are likely to develop a stronger sense of loneliness and to suffer from poorer QOL (Teo, 2010).

Despite such negative impacts of the disorder, the majority of teenagers with SAD are likely to be untreated. Psychotropic medication and CBT are the most common therapeutic options for SAD. However, socially anxious teenagers rarely seek help due to the potential stigma associated with mental issues and fear of interacting therapists and psychiatrists (Ogino, 2004; Teo, 2010). As revealed by the follow-up conducted in the current study, many of the participants treated with CBD became positive in their attitude toward seeking treatment. To overcome the dilemma of teenagers with SAD described above, delivering interventions with CBD could be an effective option for reducing the barriers facing SAD patients in need of treatment.

In all, the results of the current study provide evidence for anxiolytic effects of repeated CBD administration in teenagers with SAD. At the same time, however, the author acknowledges several limitations of the current study. No assay of the blood level of CBD was undertaken. A more detailed baseline sociodemographic evaluation could have been performed to ensure the pretreatment similarity of the treatment groups. Measurements need to be performed at additional times between the baseline and the end of the study. These measures would be essential to show, for example, if CBD could produce rapid improvement of social anxiety (a putative advantage over paroxetine). Moreover, possible side effects should be evaluated systematically. Clearly, these are issues for future research that should also be long-term studies with a positive control (e.g., paroxetine) to better assess the potential usefulness of CBD in the therapy of SAD.

## Data Availability Statement

All datasets generated for this study are included in the article/supplementary material.

## Ethics Statement

The studies involving human participants were reviewed and approved by the Institutional Ethics Committee of Kyoto University (#2018-150), the Regional committee for medical and health research ethics (#2018/1783), and the Japanese Data Inspectorate for clinical trials (JCT0018004564). The patients/participants provided their written informed consent to participate in this study.

## Author Contributions

NM conceived the study, collected and analyzed the data, and drafted the manuscript.

### Conflict of Interest

The author declares that the research was conducted in the absence of any commercial or financial relationships that could be construed as a potential conflict of interest.
